# Combination of rapamycin and SAHA enhanced radiosensitization by inducing autophagy and acetylation in NSCLC

**DOI:** 10.18632/aging.203226

**Published:** 2021-07-28

**Authors:** Yong Wang, Fen Liu, Chen Fang, Liyao Xu, Lin Chen, Zeyao Xu, Jiaquan Chen, Wei Peng, Biqi Fu, Yong Li

**Affiliations:** 1 Department of Medical Oncology, The First Affiliated Hospital of Nanchang University, Nanchang 330000, China; 2 Critical Care Medicine, The First Affiliated Hospital of Nanchang University, Nanchang 330000, China; 3 Department of Paediatrics, Children's Hospital, Zhejiang University School of Medicine, Hangzhou 310000, China; 4 Department of Internal Neurology, Jiangxi Provincial People's Hospital, Nanchang 330000, China; 5 Department of Rheumatology, The First Affiliated Hospital of Nanchang University, Nanchang 330000, China

**Keywords:** autophagy, DNA damage, histone deacetylase inhibitor, radiation, rapamycin

## Abstract

Radiotherapy plays an essential role in the treatment of non-small-cell lung cancer (NSCLC). However, cancer cells' resistance to ionizing radiation (IR) is the primary reason for radiotherapy failure leading to tumor relapse and metastasis. DNA double-strand breaks (DSB) repair after IR is the primary mechanism of radiotherapy resistance. In this study, we investigated the effects of autophagy-inducing agent, Rapamycin (RAPA), combined with the histone deacetylase inhibitor (HDACi), Suberoylanilide Hydroxamic Acid (SAHA), on the radiosensitivity of A549 and SK-MES-1 cells, and examined the combination effects on DNA damage repair, and determined the level of autophagy and acetylation in A549 cells. We also investigated the combination treatment effect on the growth of A549 xenografts after radiotherapy, and the level of DNA damage, autophagy, and acetylation. Our results showed that RAPA combined with SAHA significantly increased the inhibitory effect of radiotherapy compared with the single treatment group. The combined treatment increased the expression of DNA damage protein γ-H2AX and decreased DNA damage repair protein expression. RAPA combined with SAHA was induced mainly by regulating acetylation levels and autophagy. The effect of combined treatment to increase radiotherapy sensitivity will be weakened by inhibiting the level of autophagy. Besides, the combined treatment also showed a significantly inhibited tumor growth in the A549 xenograft model. In conclusion, these results identify a potential therapeutic strategy of RAPA combined with SAHA as a radiosensitizer to decreased DSB repair and enhanced DNA damage by inducing acetylation levels and autophagy for NSCLC.

## INTRODUCTION

Lung cancer is primary cancer and the leading cause of cancer-related death across the globe [1]. The incidence and mortality rates of lung cancer were still high, whether around the world or in China [2, 3]. Approximately 85% of lung cancers are non-small-cell lung cancer (NSCLC), and patients tend to be diagnosed as locally advanced and advanced [4]. As complete resection of the lesion cannot be achieved for surgery treatment or the indications for surgery have been lost, radiotherapy has become one of the main essential methods for treatment [5]. Despite the tremendous advances and progress made in radiotherapy research for NSCLC, the effect of treatment was still not satisfactory as radiation resistance. Relapse and metastasis still occurred in a short time after radiotherapy leading to treatment failure. Therefore, discovering effective therapeutic strategies to increase radiation sensitivity are necessary to be developed for NSCLC patients.

DNA double-strand breaks (DSB) are the most severe form of DNA damage response after radiation and are a primary mechanism to kill cancer cells for radiotherapy [6]. However, DSB repair will be efficiently activated and repaired after IR and limits the effectiveness of radiotherapy. Cells will stop dividing and turn into a brief moment cell cycle arrest to DSB repair. Two major DSB repair pathways, homologous recombination (HR) and non-homologous end-joining (NHEJ), participated in DNA damage repair after radiotherapy [7, 8]. Multiple DSB damage repair proteins, such as Rad51, DNA-PKcs, Ku70, and Ku80, are involved in the repair process [9–11]. It has been demonstrated that Rad51, the critical proteins in the HR pathway, and Ku70, Ku80, the critical proteins in the NHEJ pathway, played a critical role in DSB repair induced by radiotherapy [9, 10]. Increased DSB repair proteins result in radiation resistance and tumor therapies [12]. Dysregulation of the DSB repair pathway is associated with cancer development and is the new target for tumor treatment strategies. Targeting the DSB repair pathway by regulating the core components in the DSB repair pathway to counteract radiation resistance is a potential approach and promising strategy for NSCLC treatment [13].

Autophagy, a multi-step lysosomal degradation process of dysfunctional cellular elements and organelles that maintains cellular metabolic balance, has been reported that plays a vital role in regulating cancer progression by coping with environmental stresses [14]. Growing evidence has shown that tumor resistance to radiotherapy can be improved by regulating the level of autophagy in the cancer cell. Deville SS et al. reported that Keap1 inhibition enhanced head and neck squamous cell carcinoma (HNSCC) cells radiosensitivity by inducing autophagy to influence the process of DNA damage repair [15]. The level of autophagy in MCF7 breast cancer (BC) cells can be increased by mTOR inhibitor, rapamycin (RAPA), delayed the attendance of Rad51 and BRCA1 protein localization, and prolonged the expression of damage characteristic sensor γ-H2AX, resulted in the accumulation of DSB damage [16]. In contrast, Ning et al. demonstrated that suppression of autophagy increased the IR sensitivity of nasopharyngeal carcinoma (NPC) cells by reducing the expression of Rad51. That indicated autophagy has a dual role in the radiotherapy of tumors. Regulation of autophagy in different strategies for cancer cell death or survival is dependent on the cancer context. Our previous study showed that stimulating autophagy by RAPA can downregulation expression of Rad51 and Ku80, delayed DNA damage repair, and enhanced the degree of damage DSB in NSCLC A549 cells [17]. Reducing the protein expression of the DSB repair pathway by regulating autophagy can improve radiotherapy response. However, autophagy formed in the cytoplasm can only affect the DSB repair protein in the cytoplasm and fails to effectively degrade the proteins recruited for DSB repair in the nucleus. Higher levels of autophagy may be needed to induce autophagic death of tumor cells to achieve the purpose of radiosensitization. However, toxic and side effects, autophagic death, on normal cells will also increase as the dose of the drug increases to induce autophagy. Therefore, degrading DSB repair proteins in the nucleus is the key to enhance the radiosensitivity of NSCLC further.

Increasing evidence in large numbers of studies has shown that histone deacetylases (HDACs) are highly expressed in many kinds of tumors, making the intracellular histones in a state of deacetylation [18]. Histone deacetylase inhibitor (HDACi) is a new generation of anticancer drugs based on epigenetics. These drugs target HDACs and exert anti-tumor effects by regulating the acetylation levels of histones and related non-histone proteins, such as critical related proteins for DNA damage recognition and repair [19–22]. Robert et al. explicitly stated that HDACi Valproic acid (VPA) affects the DSB repair process by degrading acetylated DSB repair recombination protein through autophagy in yeast cells [23]. When some specific proteins are hyperacetylated, they will shunt into the autophagic pathway [24]. It confirmed that HDACi can influence the process of the DSB repair pathway at low concentrations in tumor cells and has little effect on normal cells [25, 26]. HDACi can induce autophagy in tumor cells [27], but the ability to induce autophagy is not strong [23].

Therefore, we hypothesized that the induction of autophagy combined with the acetylation effect of HDACi on the DSB repair pathway could enhance the radiosensitivity of NSCLC cells by degrading the recombination repair protein through autophagy. In the present study, we investigated the combined effect of suberoylanilide hydroxamic acid (SAHA) and RAPA on the radiosensitivity of NSCLC cells. We elucidated the role related to affect the process of the DSB repair pathway by further degrading the expression of Rad51, Ku70, and Ku80 repair protein.

## RESULTS

### RAPA or/and SAHA induced cytotoxicity in NSCLC cells

**Figure 1:**
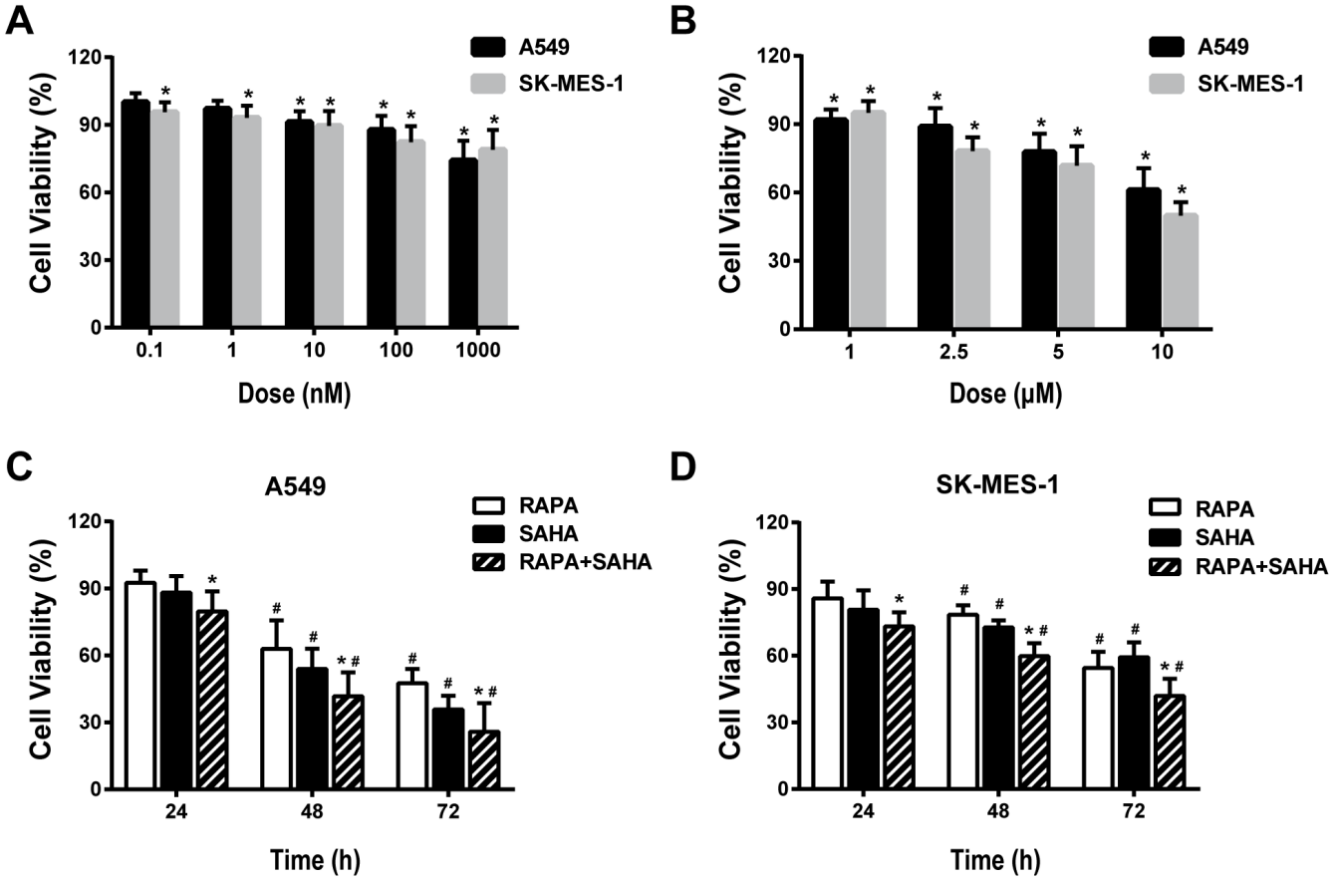
**Cytotoxic effects of combination treatment with RAPA or/and SAHA in NSCLC cells.** Cell viabilities were assessed by the cell counting kit-8 (CCK-8). (**A**, **B**) Concentration-dependent effects of RAPA (**A**) and SAHA (**B**) on the viability of two NSCLC cells (A549, SK-MES-1) for 24h. *p<0.05, RAPA or SAHA versus control. (**C**, **D**) Time-dependent effects of RAPA (100nmol/L) or/and SAHA (2.5μmol/L) on the viability of A549 (**C**) and SK-MES-1 (**D**) cells. *p<0.05, combined treatment versus single treatment, ^#^*p*<0.05, 48h or 72h versus 24h.

The viability of cultured A549 and SK-MES-1 cells was measured by CCK8 assay after 24 hours of exposure to RAPA or SAHA at different concentrations. RAPA and SAHA suppressed NSCLC cell proliferation in concentration-dependent manners (Figure 1A, 1B). The viability of NSCLC cells exposure to 100nM of RAPA or 2.5μM of SAHA alone or in combination was observed at various time points. RAPA and SAHA reduced the viability of NSCLC cells in time-dependent manners. The combined treatment of RAPA and SAHA compared with single-drug treatment alone were significantly enhanced toxicity in A549 and SK-MES-1 cells for 24, 48, and 72 hours (Figure 1C, 1D).

### Combination treatment of RAPA and SAHA enhances the radiosensitivity in NSCLC cells

**Figure 2:**
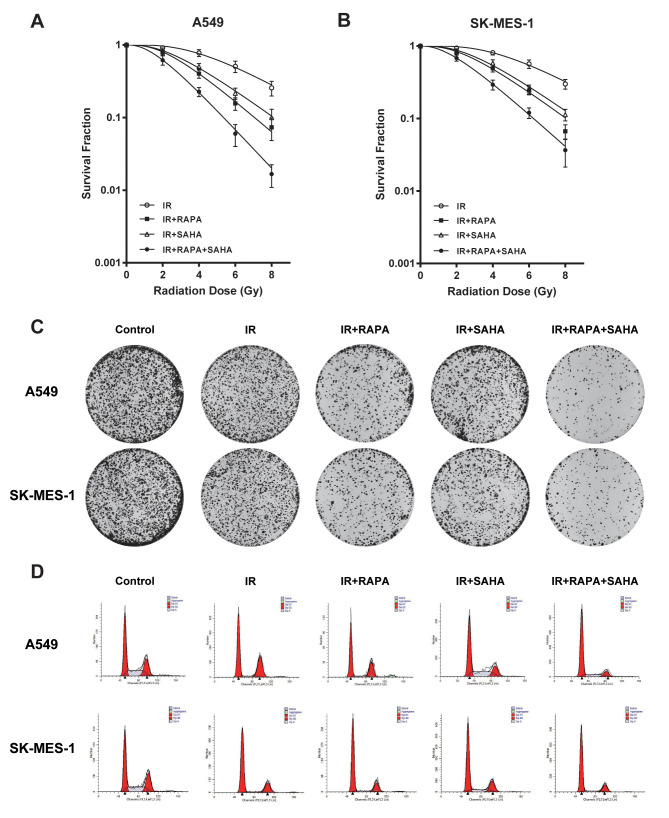
**Cytotoxic effects of combination treatment with RAPA or/and SAHA in irradiated NSCLC cells.** Survival fractions were assessed by the colony formation assay. (**A**, **B**) The radiation dose-response survival curves after 24h of treatment with RAPA (100nmol/L) or/and SAHA (2.5μmol/L) in two NSCLC cells A549 (**A**), SK-MES-1 (**B**). (**C**, **D**) Colony formation assay (**C**) and flow cytometry analysis of the cell cycle (**D**) in two NSCLC cells (A549, SK-MES-1) resulting from IR (4Gy) after treatment with RAPA (100nmol/L) or/and SAHA (2.5μmol/L) for 24h.

In our preview study, we showed that RAPA treatment sensitized A549 cells to radiation [17]. To investigate whether SAHA may coordinate with RAPA on radiosensitivity in NSCLC cells, clonogenic assays were performed to evaluate the combined effect of RAPA and SAHA in A549 and SK-MES-1 cells on IR. As 100nM of RAPA and 2.5μM of SAHA had modest effects on viability in the two NSCLC cell lines for 24 hours (Figure 1), NSCLC cells were treated with RAPA (100nM) and SAHA (2.5μM) for 24 hours and subsequently irradiated cells with different dose of γ-irradiation. Combination treatment of RAPA and SAHA resulted in higher IR induced cytotoxic effects in A549 and SK-MES-1 cells and significantly reduced the survival fraction in a dose-dependent manner than those of IR treatment alone or in combination RAPA or SAHA (Figure 2A, 2B). Compared with IR alone or in combination with RAPA or SAHA, combination treatment of RAPA and SAHA significantly increased IR-induced clonogenic cell death in both A549 and SK-MES-1 cells with 4Gy of γ-irradiation (Figure 2C).

Our data showed that IR induced G2/M phase arrest in A549 and SK-MES-1 cells. G0/G1 phase arrest were induced by pre-treatment with RAPA before IR, and S phase arrest were induced by pre-treatment with SAHA. Combination treatment of RAPA and SAHA led to cell cycle arrest in the G0/G1 phase (Figure 2D).

### Effect of RAPA and SAHA combination treatment on DNA damage and DNA repair in NSCLC cells with IR treatment.

**Figure 3:**
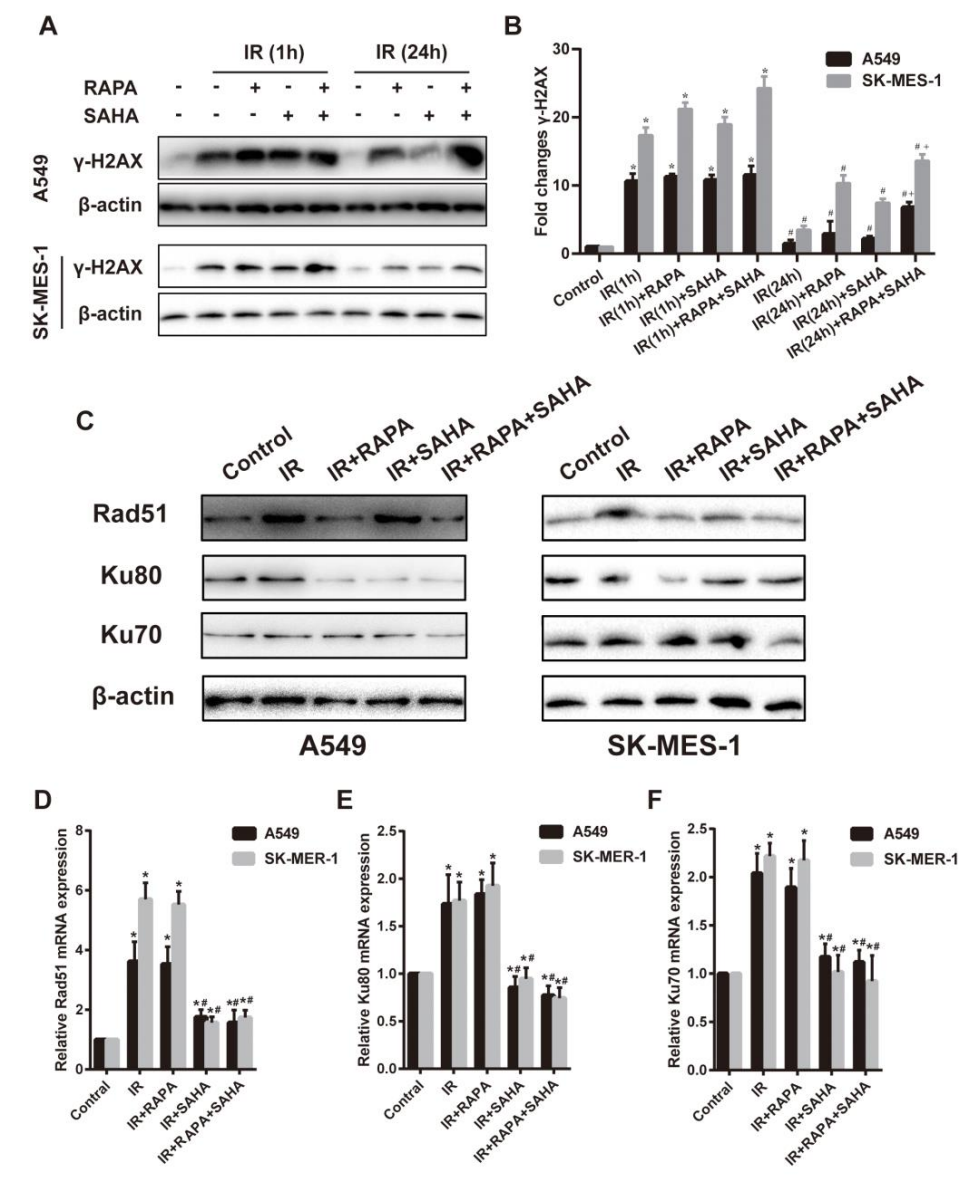
**Effects of combination treatment with RAPA and SAHA on DNA damage and repair after IR in NSCLC cells.** (**A**, **B**) the protein level of γ-H2AX was determined by western blot analysis. Two NSCLC cells (A549, SK-MES-1) were treated with RAPA (100nmol/L) or/and SAHA (2.5μmol/L) for 24h and were subsequently exposed to IR (4Gy), the γ-H2AX protein was tested at 1h and 24h after IR. *p<0.05, 1h after IR versus control; ^#^*p*<0.05, 24h versus 1h after IR; ^+^*p*<0.05, combined treatment versus single treatment 24h after IR. (**C**–**F**) the protein (**C**) and mRNA level of Rad51 (**D**), Ku80 (**E**), Ku70 (**F**) were determined by western blot and RT-qPCR analysis. Two NSCLC cells (A549, SK-MES-1) were treated with RAPA (100nmol/L) or/and SAHA (2.5μmol/L) for 24h and were subsequently exposed to IR (4Gy) and were tested after 4h. *p<0.05, compared with control, ^#^*p*<0.05, compared with IR.

Variant H2AX was immediately phosphorylated (γ-H2AX) and accumulated at DSB sites after IR. The expression of γ-H2AX could be an indicator of the degree of DNA damage. Our results showed that γ-H2AX significantly increased at 1 hour after IR compared to controls in both A549 and SK-MES-1 cells (Figure 3A, 3B), regardless of RAPA or SAHA treatment alone or in combination treatment. IR induced γ-H2AX gradually decreased after 24 hours, almost back to the original expression level. However, the combination treatment of RAPA and SAHA significantly enhanced the level of γ-H2AX compared with IR alone or in combination with RAPA or SAHA at 24 hours after treatment (Figure 3A, 3B).

Cancer cells could repair DSB through HR and NHEJ repair pathways. DNA repair-related proteins, such as Rad51, Ku80, and Ku70, are essential players in the two pathways. Results showed that compared with the IR group, the combination treatment of RAPA and SAHA had been found obviously to downregulate DNA repair-related proteins, such as Rad51, Ku80, Ku70 in A549 cells, and Rad51, Ku70 in SK-MES-1 cells. Treatment with RAPA alone, Rad51 and Ku80 were decreased in both A549 and SK-MES-1 cells; or treatment with SAHA alone, Ku80 were decreased in A549 cell, and Rad51 were decreased in SK-MES-1 cells. (Figure 3C–3F).

Our results showed that IR increased the mRNA expression level of DNA repair-related proteins, Rad51, Ku80, and Ku70, detected by RT-qPCR. Treatment with SAHA alone or combination treatment of RAPA and SAHA, mRNA of Rad51, Ku80, and Ku70 decreased in NSCLC cells compared with IR. However, mRNA expression of all DNA repair-related proteins was unchanged in RAPA compared to IR.

### Effect of RAPA or SAHA alone or in combination on autophagy and acetylation in NSCLC cells with IR treatment

**Figure 4:**
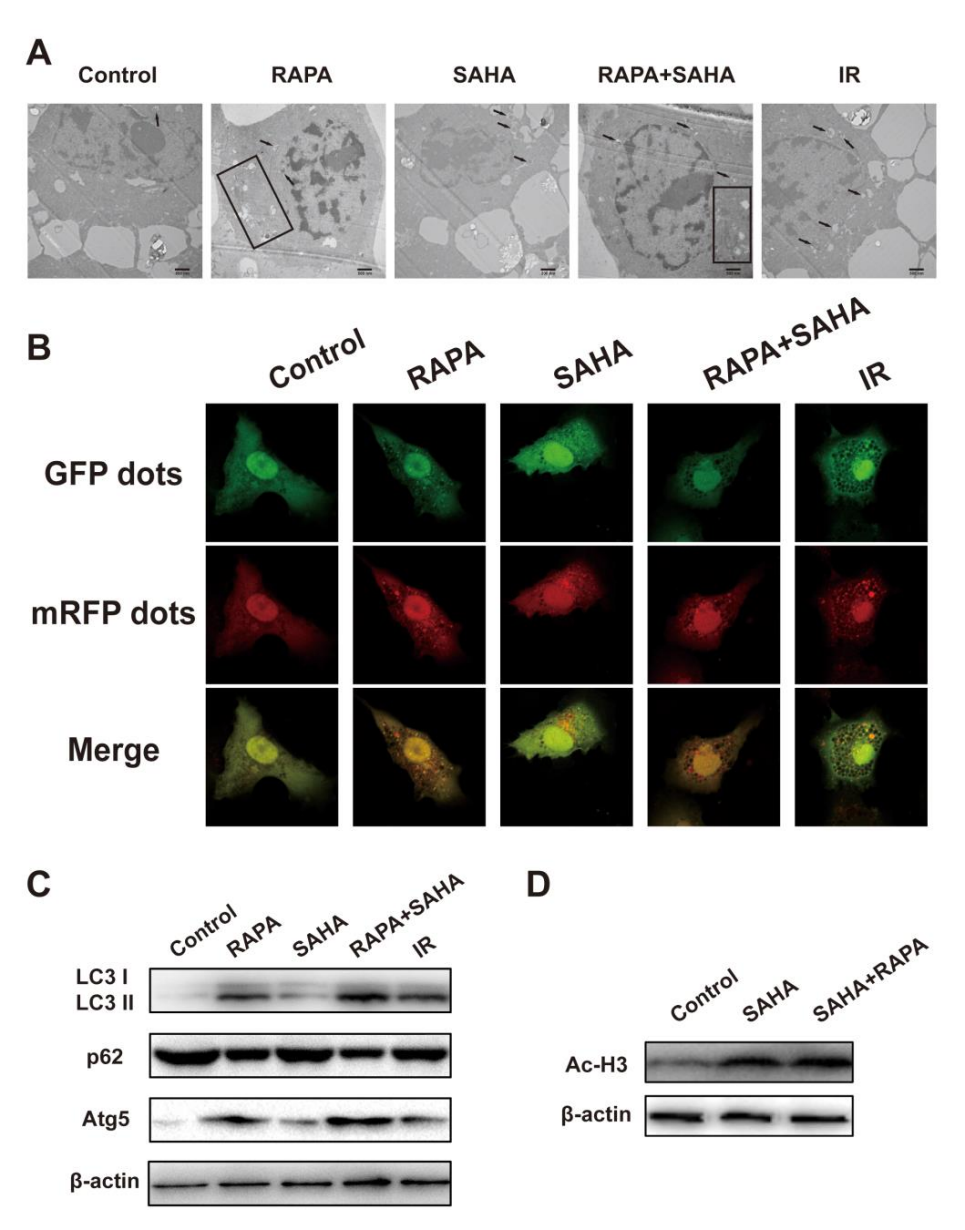
**Effects of combination treatment with RAPA and SAHA on autophagy and acetylation in NSCLC cells.** (**A**–**C**) RAPA, SAHA, and IR induce cellular autophagy. A549 cells were treated with RAPA (100nmol/L) or/and SAHA (2.5μmol/L) for 24h or were exposed to IR (4Gy) and were tested after 4h. (**A**) The ultrastructures of autophagosomes in A549 cells were observed under a transmission electron microscope (TEM). Black arrows and rectangles indicate intracellular autophagosomes. (**B**) The distribution of LC3 dots in A549 cells was observed by using an immunofluorescence confocal microscope. Quantitative data were calculating the number of LC3 dots per cell. *p<0.05, compared with control, ^#^*p*<0.05, compared with combined treatment. (**C**) The level of autophagy-related protein (LC3 I/II, p62, Atg5) was determined by western blot analysis. (**D**) SAHA induces acetylation of A549 cells. A549 cells were treated with SAHA (2.5μmol/L) and/or RAPA (100nmol/L) for 24h. The level of acetylation of histone H3 was determined by western blot analysis.

Our previous studies have demonstrated that RAPA induces autophagy sensitizes A549 cells to IR. To determine whether combined treatment can be induced autophagy and acetylation in A549 cells. Electron microscopy shows that IR, RAPA, SAHA, and combined treatment could induce the formation of autophagosomes in the cytoplasm of A549 cells (Figure 4A). Compared with other groups, combined treatment primarily increased the number of autophagosomes. We applied confocal immunofluorescence microscopy to detect the dots of LC3, which is widely used as a marker of autophagy in per A549 cells. By quantitative analysis, dots of LC3 were significantly increased in the cytoplasm of A549 cells after combined treatment compared with each of the different treatments (Figure 4B). LC3-II, Atg5, and p62 proteins are the most common autophagic-related markers. Our results revealed that combined treatment remarkably increased the expression of LC3-II and Atg5 proteins and marked decreased the expression of p62 proteins compared with RAPA, SAHA, or IR treatment alone (Figure 4C). The result indicated that both RAPA, SAHA, and IR could induce autophagy. The effect of RAPA is most significant.

Ac-H3 was chosen as a marker to monitor the effect of HDAC inhibition. As shown in Figure 4D, the expression of Ac-H3 protein was increased after exposure to SAHA alone or RAPA combined with SAHA. But no difference between the two treatments. These results indicated that only SAHA, not RAPA, has the effect of acetylation of histone and non-histone proteins.

### Inhibition of autophagy decreases the sensitivity of NSCLC cells to IR

**Figure 5:**
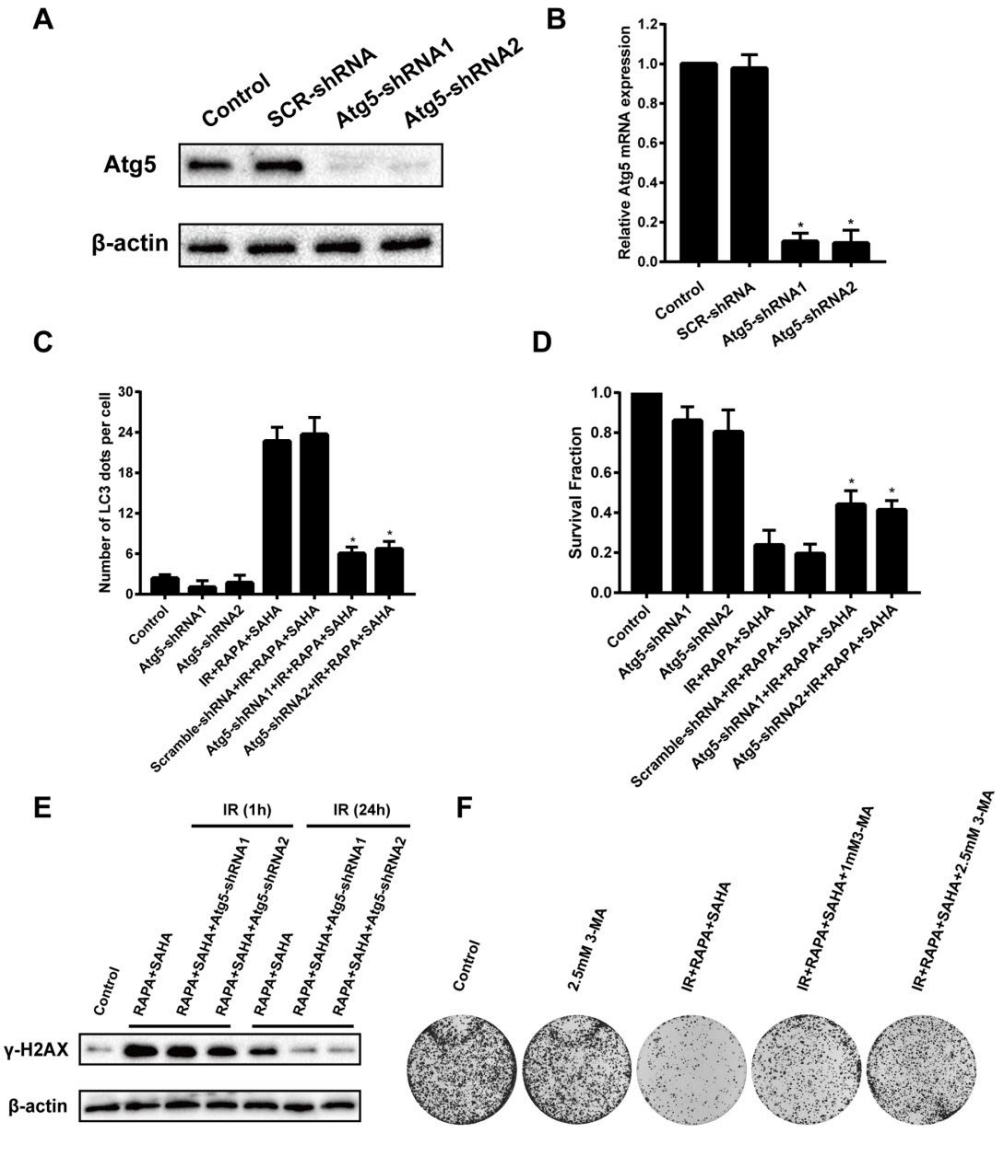
**Effects of combination treatment with RAPA and SAHA on IR after down-regulate the level of autophagy in NSCLC cells.** A549 cells were infected by a lentivirus delivered Atg5 shRNA for 24h. (**A**, **B**) The relative protein and mRNA expression levels of Atg5 were determined by western blot and RT-qPCR analysis. (**C**) Quantitative data were calculating the number of LC3 dots per A549 cell by using an immunofluorescence confocal microscope. (**D**) Survival fractions of A459 cells were assessed by the colony formation assay. (**E**) γ-H2AX protein was determined by western blot analysis. A549 cells were treated with RAPA (100nmol/L) and SAHA (2.5μmol/L) for 24h and were subsequently exposed to IR (4Gy) for 1h and 24h after transfected with or without Atg5 shRNA for 24h. (**F**) Colony formation assay in A549 cells resulting from IR after treatment with different concentrations of 3-MA or without 3-MA. A549 cells were pretreated with 3-MA for 1h before RAPA and SAHA treatment.

To confirm the function of autophagy in combined treatment-induced cytotoxicity after IR, A549 cells were transfected with Atg5 shRNA to induce Atg5 gene silencing then treated with RAPA and SAHA. Atg5 protein and mRNA expression were markedly decreased in A549 cells by transfected with Atg5-shRNA1 or Atg5-shRNA2 construct virus (Figure 5A, 5B). Besides, we found that the LC3 dots were remarkably decreased by transfected with Atg5-shRNA compared with the control shRNA (Figure 5C). This means that the effect of inducing autophagy by combined treatment of RAPA, SAHA, and IR will be attenuated by down-regulation of Atg5. Furthermore, inhibition of autophagy by transfected with Atg5-shRNA reduced the death of A549 cells after IR was treated with the combined treatment (Figure 5D). We have found that the combination treatment significantly enhanced the level of γ-H2AX after 24 hours of IR. The expression of γ-H2AX after 24 hours IR was markedly decreased by transfected with Atg5-shRNA (Figure 5E). We also used autophagy inhibitor 3-MA to inhibit the level of autophagy from testing the role of autophagy in radiotherapy. Our results showed that combined with 3-MA significantly reduces the efficacy of combined treatment of RAPA and SAHA for radiosensitization by clonogenic assays (Figure 5F). The results indicated that down-regulate autophagy conducive to DNA damage repair after IR, which led to reduce DNA damage and radiosensitization in cancer cells.

### Effects of combination treatment with RAPA and SAHA enhances the anti-tumor effects of IR in an NSCLC model

**Figure 6:**
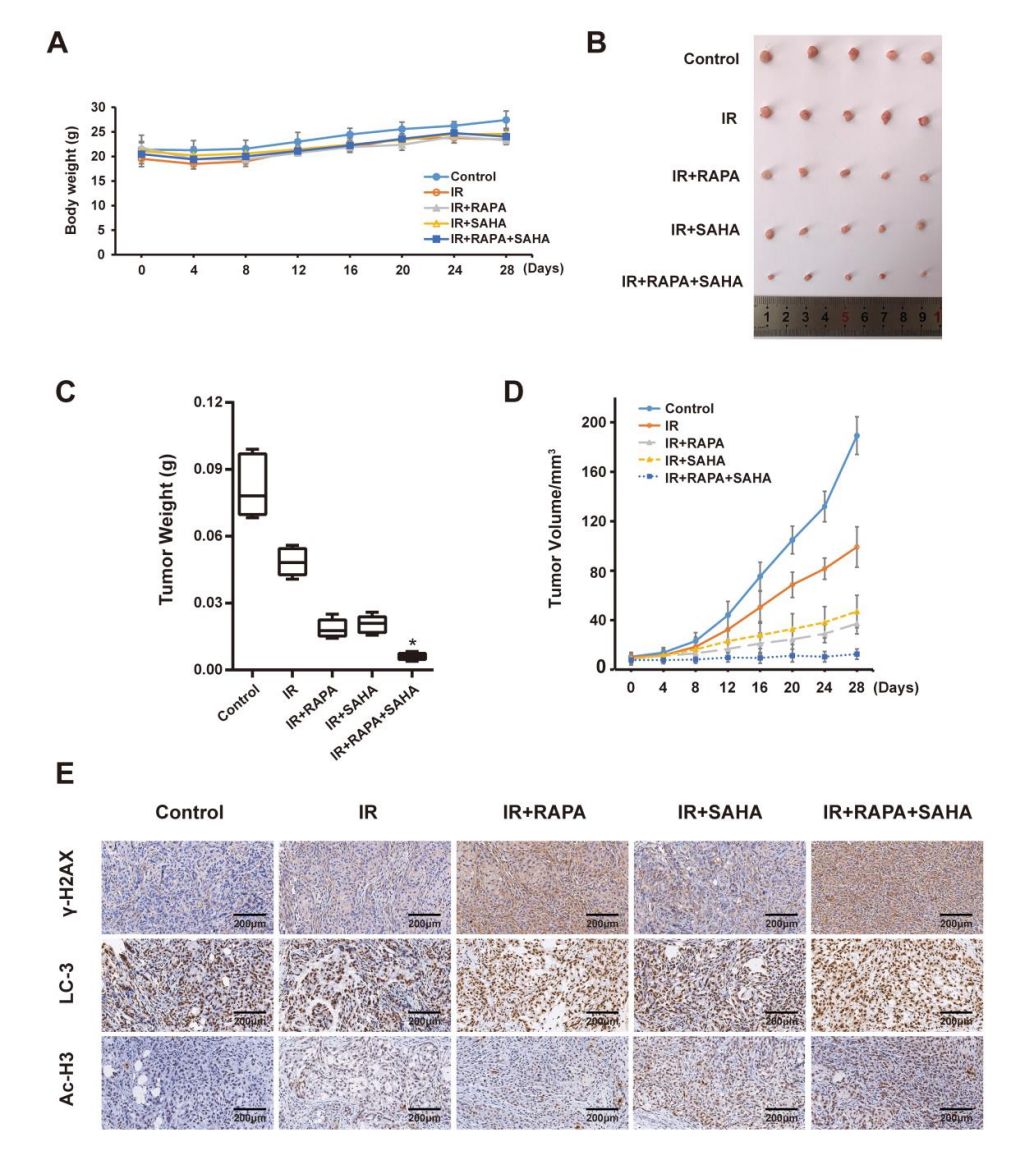
**Effects of combination treatment with RAPA and SAHA on A549 xenografts model after receiving IR.** (**A**) Bodyweight in A549 cell xenografts measured every 4 days. (**B**, **C**) Specimens and tumor weight of A549 cell xenograft after different treatments for 28 days. (**D**) Tumor volume of A549 cell xenograft in nude mice measured every 4 days. (**E**) Immunohistochemical staining of γ-H2AX, LC3 and Ac-H3 in A549 cell xenografts (×200).

We next examined the anti-tumor effect of RAPA and SAHA either alone or in combination treatment in the A549 xenografts model after receiving IR *in vivo*. The body weight and tumor volume of the mice were measured every four days. Our results showed that the bodyweight of each group slightly decreased in the short term of treatment, then bodyweight gradually increased (Figure 6A). It indicated that each of the interventions does not produce toxic side effects based on the weight of mice. By measuring the tumor weight of each group after 28 days of intervention, we found that combined treatment of RAPA and SAHA dramatically inhibits the growth of the tumor compared with IR or other treatments (Figure 6B, 6C). We further measured the change of tumor volume every four days, the tumor volume in each group was gradually increased in a time-dependent manner, the inhibition effect of combined treatment on tumor volume was marked compared with other groups. Finally, we examined the expression of γ-H2AX, LC3, and Ac-H3 in the A549 tumor. The expression of γ-H2AX and LC3 were increased after IR compared with the control group. Tumor tissues from the combined treatment of RAPA and SAHA showed higher c-H2AX and LC3 than the tumor tissues from IR with RAPA or SAHA or IR alone. The expression of Ac-H3 in tumor tissues was increased in IR with SAHA and combined treatment. Results indicated that combined treatment of RAPA and SAHA significantly up-regulated autophagy and acetylation increased the DNA damage in A549 xenograft models.

## DISCUSSION

Radiotherapy has become one of the common strategies for advanced lung cancer (III and IV). However, radiotherapy technology's tremendous improvement and progress, such as intensity-modulated radiotherapy (IMRT) and three-dimensional conformal radiation therapy (3D-CRT), resistance to IR of tumor cells leads to poor radiotherapy treatment outcomes NSCLC. Intracellular signaling pathways promote radiotherapy resistance and tumor cell survival by activating the classic DSBs repair pathways, HR and NHEJ. New strategies of treatment to overcome tumor radioresistance are urgently needed to be ameliorated.

Our previous study showed that RAPA sensitized A549 cells to radiation by inducing autophagy [17], but excessively regulating the autophagy level will damage normal cells, autophagic death will be followed [28]. More and more evidence revealed that with double synergistic effects, anti-tumor drugs, HDACi, affected radiosensitivity of multiple types of tumors [29–31]. A study reported that many kinds of HDACi, FK228, TSA, VPA, and SAHA, could enhance radiation sensitivity by inducing G1 phase arrest in melanoma cells [32]. In the present study, we found that the treatment of RAPA combined with SAHA can increase the radiation sensitivity of NSCLC both *in vitro* and *in vivo* (Figures 2, 6). With the increasing dosage of drugs, both RAPA, SAHA, or combined treatment, and the extension of intervention time, it has a specific inhibitory effect on the growth of NSCLC cells (Figure 1). It has been confirmed that HDACi with lower toxic-side effects on normal cells at low concentrations and a broad anti-cancer spectrum [26, 33].

Moreover, the bodyweight of RAPA and SAHA treated A549 cancer cells xenografts model were no apparent changes (Figure 6A). That indicated that no conspicuous observable toxicity was detected *in vivo*. Therefore, SAHA combined with RAPA is a potential security and effective treatment strategy to enhances radiosensitivity in NSCLC.

The cell cycle process will be changed by the mechanism of monitoring points when the DNA is damaged by radiation. Cells in different phases of the cell cycle affect radiosensitivity. Cells in G2/M phases are most radiosensitive, less sensitive in the G1 phase, and most radioresistant in the S phase [34]. However, not as our expectation, we found that combination treatment of RAPA and SAHA led to cell cycle arrest in G0/G1 phase arrest, not in the most radiosensitive, G2/M (Figure 2D). Therefore, the mechanism to increase radiosensitivity did not entirely involve cell cycle arrest.

By increasing the DNA damage of malignant cells after IR, especially DSB, which is considered as the most critical type of DNA damage [35], and affecting the DSB repair pathway, HR and NHEJ, after the damage is the primary mechanism for hypersensitivity to radiation in series of publications [8, 31, 36]. We detected the protein expression level of γ-H2AX in NSCLC cells. The level of γ-H2AX was significantly increased in all groups at 1 hour after IR and was gradually decreased at 24 hours after exposure to IR. However, a higher expression level of γ-H2AX was detected 24 hours after IR in RAPA and SAHA combined treatment compared with other groups (Figure 3A, 3B). The expression level of γ-H2AX was also significantly enhanced in the combination of RAPA and SAHA group after IR *in vivo* by the A549 xenografts model. Results indicated that RAPA combined with SAHA prolonged the damage of NSCLC cells after IR. Rad51 protein has been demonstrated to be a critical molecule in the HR repair pathway and Ku80 and Ku70 in the NHEJ repair pathway. It was reported that SAHA and cisplatin treatment in combination with IR suppressed the expression level of rad51 protein in NSCLC cells and Ku80 in H460 cells [37]. SAHA attenuated the expression of Rad51 and DNA-PK repair proteins in BC 4T1 cells [38]. Our study found that suppression of HR and NHEJ repair-associated proteins (Rad51, Ku80, and Ku70) in RAPA and SAHA combined treatment after IR. Results indicated that combined treatment increased DSB after radiation by reducing the protein expression in both the HR and NHEJ repair pathways.

A series of literature has been reported that autophagy, the process of lysosomal-mediated cellular self-digestion, plays a critical role in tumor therapy. The effects of autophagy in the development of radiotherapy have been widely researched [39, 40]. Numerous previous research has shown that the pro-death role of autophagy. Daido et al. indicated that glioblastoma multiforme cells could be radiosensitizers by inducing autophagy [41]. However, autophagy can usually be detected after radiotherapy and related to radioresistance, and it can increase radiosensitivity by inhibiting autophagy [41]. Therefore, whether autophagy in radiotherapy was a cytoprotective or disadvantageous factor for cancer cells remains controversial [39]. Our results, consistent with previous reports [40, 42–44], demonstrated that both IR, SAHA, and RAPA induced autophagy of NSCLC cells. We also showed that RAPA enhanced a remarkable level of autophagy compared with IR and SAHA (Figure 4A–4C). A previous study explicated that some specific hyperacetylated proteins were inclined to enter the autophagic pathway [24]. We demonstrated that RAPA and SAHA treated NSCLC cells showed a lower DSB repair protein expression and durable DNA damage protein, γ-H2AX, expressed after IR and hypersensitivity to IR (Figures 3A–3C, 2B). So we tested the acetylation level of cancer cells after treated with RAPA and SAHA. Our findings showed that SAHA, not RAPA, upregulated acetylation of A549 cells (Figure 4D). Induction of autophagy and acetylation could also be further tested *in vivo* by the A549 cells xenografts model (Figure 6E). We further strengthened the role of autophagy in radiotherapy by transfected with the target Atg5 shRNA (Figure 5A, 5B) or pre-treated with 3-MA and then exposed to RAPA, SAHA, and IR. Results indicated that inhibition of autophagy by Atg5 shRNA resulted in weakening the effect of RAPA combined with SAHA on radiotherapy (Figure 5D) and reduced the expression of γ-H2AX (Figure 5E). Clonogenic assays demonstrated that pretreated with 3-MA on different concentrations reduced the toxicity of IR increased by RAPA and SAHA as the level of autophagy decreased (Figure 5F). Therefore, results indicated that RAPA and SAHA treatment could further enhance NSCLC cell death by inducing autophagy to affect the repair process of DSB and increase DNA damage after IR. However, the exact mechanisms of autophagy affecting DNA damage repair proteins are unclear. Further researches are needed to be elucidated in the future.

In summary, our data demonstrate that the combination of RAPA with SAHA exhibited more potent cytotoxicity and enhanced the radiosensitivity of NSCLC *in vitro* and *vivo* by affected DSB repair pathway and prolonged the DNA damage induced by IR. It was suggested that a higher acetylation level induced by SAHA might promote the degradation of repair proteins and that autophagy induced by RAPA may be involved in the process of degradation may be one of the underlying mechanisms. Therefore, more detailed studies are required to explicate the relationship between autophagy and the DSB repair pathway.

## MATERIALS AND METHODS

### Cell culture and irradiation treatment

The human NSCLC cell lines A549 and SK-MES-1 cells, purchased from the Type Culture Collection of the Chinese Academy of Sciences, Shanghai, China, were both grown in high glucose DMEM medium (BI, Israel) supplemented with 10% fetal bovine serum (Gibco, Grand Island, USA), penicillin (100 U/ml), and streptomycin (100 μg/ml). The cells were incubated in a humidified atmosphere containing 5% CO^2^ at 37° C. IR was operated with 6 MV X-rays using a linear accelerator (Digital Precise Accelerator, Elekta Infinity, Sweden) at a 4 Gy/min dose rate.

### Cell viability assay

Cells were seeded in 96-well plates at a density of 5×10^3^ cells/well and adhered to the wall for 24h at 37° C. Cells were then treated with RAPA (100nM) or/and SAHA (2.5μM). Subsequently, cell counting kit-8 (CCK-8) agentia (Abmole Bioscience, USA) 10μL was added to each well, and plates were hatched for 2-4h at 37° C, abide by the CCK-8 kit protocol. Optical density (OD) values were measured at 450nm using a plate reader (Wyatt Technology Corporation, USA). The average cell proliferation inhibition rate was calculated as (OD of the control group – OD of the experimental group)/(OD of the control group – OD of the blank group)×100%.

### Clonogenic survival assay

The NSCLC cells were seeded into wells of a six-well plate with a concentration of 1000 cells per well. After overnight incubation, cells were treated with RAPA(100nM) or/and SAHA (2.5μM) for 24h in the presence or absence of radiation administered. The culture medium was renovated every three days. Let all cells grow for another 14 days to form colonies, then were stained with 0.1% crystal purple. Colonies with more than 50 cells were counted. The clone formation rate (PE) and survival rate (SF) were calculated. PE (%) = number of clones / number of inoculated cells × 100%. SF=PE combined group / PE simple irradiation group × 100%. The average will be obtained by using the Sigmaplot software, according to the multi-target single. The cell survival curve was fitted by hitting the model Y=1-(1-exp(-x/D0))^N^, and the cell survival curve was calculated.

### Cell cycle analysis by flow cytometry

According to the instructions, cell-cycle arrest profiles were analyzed by the Cell cycle staining commercial Kit (MultiSciences Biotech Co, Ltd., Hangzhou, China). The NSCLC cells were harvested and then washed once with PBS. Then incubated in 1 ml DNA staining solution and 10 μl permeabilization solution for 30 min at room temperature in the dark. Finally, the cell cycle was analyzed by flow cytometer.

### Western blot analysis

The protein lysates were isolated in 10% sodium dodecyl sulfate-polyacrylamide gel electrophoresis. Proteins were transferred onto a polyvinylidene fluoride membrane (PVDF, Merck Millipore, Darmstadt, Germany), blocked with 5% buttermilk for one hour at room temperature, and cultivated with a primary antibody overnight at 4° C. Primary antibodies used included anti-LC3, anti-Atg5 (1:5000, 1:2000, and 1:2000 dilution, Sigma-Aldrich, St. Louis, Missouri, USA), anti-p62, anti-Rad51, anti-Ku70/80, anti-β actin (1:2000, 1:1000, 1:500, and 1:1000 dilutions, singly; Abcam, ab91526, ab133534, ab53126, ab8227, Cambridge, UK), γ-H2AX (1:1000 dilution, Cell Signaling Technologies, Danvers, MA, USA), and Acetylation of histone H3 (Ac-H3) (1:2000 dilution, sc56616, Santa Cruz Biotechnology, USA). The membranes were incubated with horseradish peroxidase-conjugated secondary antibody at a dilution of 1:2000 for one hour at room temperature. Protein bands were visualized using ECL Western Blotting Detection Reagents (Thermo Fisher Scientific, RJ238937, Waltham, USA) and exposed to an ECL Plus film (GE Healthcare, Piscataway, NJ, USA), and were quantified using the ImageJ software (NIH).

### Real-time polymerase chain reaction

Total RNA was separated using Trizol (Invitrogen, Carlsbad, California, USA), according to the manufacturer’s specification. Then, we used the Superscript III First-Strand Synthesis System (Invitrogen, Carlsbad, California, USA) to compound the first-strand cDNA at a final volume of 20μL. Quantitative real-time polymerase chain reaction (RT-qPCR) analyses were processed using an SYBR Green mix in the Real-Time PCR System (TAKARA, Dalian, China). The following steps were finished according to the instructions. Human GAPDH was utilized as an internal housekeeping reference. Primers used for RT-qPCR were Rad51, Ku70, Ku80, Atg5, and GAPDH. All primers sequences were as follows: Rad51(forward primer): 5′-ATGCCAACGATGTGAAGAAA-3′; (reverse primer): 5′-CAGCTTTGGCTTCACTAATTCC-3′. Ku70(forward primer): 5′-AAAAGACTGGGCTCCTTGGT-3′; (reverse primer): 5′-TGTGGGTCTTCAGCTCCTCT-3′. Ku80(forward primer): 5′-CGACAGGTGTTTGCTGAGAA-3′; (reverse primer): 5′-GAATCACATCCATGCTCACG-3′ Atg5(forward primer): 5′-TCACAAGCAACTCTGGATGG-3′; (reverse primer): 5′- TGTGTGCAACTGTCCATCTG-3′ and GAPDH(forward primer): 5′-CTGGGCTACACTGAGCACC-3′; (reverse primer): 5′-AAGTGGTCGTTGAGGGCAATG-3′.

### Transmission electron microscope (TEM)

Cells were fixed in 4% paraformaldehyde at pH 7.4. Cells were embedded in Epon, stained with uranyl acetate, and processed as previously described. Subsequently, we observed the specimens make use of a transmission electron microscope (JEM-1400/1011, Jeol, Japan) at the core facility of Nanchang University.

### Transient transfection

We planted A549 cells into coverslip and incubated in CO_2_ incubator overnight, then were transiently transfected with ptfLC3 expressing plasmid using Lip2000 (Invitrogen 11668-027, USA) transfection system according to the manufacturer's instruction. After 24 hours, cells were managed with RAPA (100nM) or/and SAHA (2.5μM) for 24 hours in the presence or absence of radiation administered 4h. After that, cells were fixed with 4% paraformaldehyde for 15 minutes and then washed three times with PBS. Dripping medium containing DAPI to the coverslip and placing to a glass slide upside down. The localization of LC3 dots was seen and captured by a confocal microscope (×200, Leica, Wetzlar, Germany).

### Transfection of shRNA/RNA interference

Using short hairpin RNA (shRNA) (MiaolingBio, Wuhan, Hubei Province, China) to achieve Atg5 gene knockdown. The following shRNAs for Atg5 (MiaolingBio) were cloned into lentiviral vectors: 5′-GCTACTCTGGATGGGATTG-3′ for Atg5-shRNA 1; 5′-TCGTTCAGTTATCTCATCC-3′ for Atg5-shRNA 2. Lip2000 (Life Technologies) transfection reagent was used, based on the manufacturer’s instructions. In short, shRNA for Atg5, or control, scrambled shRNA was diluted into each well of a 6-well plate containing transfection medium (Sigma) and incubated for 5 minutes. Simultaneously, lipofectamine was diluted in transfection medium (Sigma) at a scale of 5μL lip2000 in 245μL transfection culture medium. The thinning lipofectamine reagent and shRNA were mixed and incubated at room temperature for 20 minutes. Cells were incubated for 24 hours after transfection. Cells were then gathered to confirm knockdown efficiency via immunoblotting and for further experiments.

### Subcutaneous xenograft models

The 3~4-week-old BALB/c nude mice, purchased from Changsha Tianqin Biotechnology Co., Ltd. (Changsha, Hunan Province, China), were maintained under a controlled temperature (22~26° C), humidity (40~70%), and 12h light/12h dark cycle. The study was approved by the Ethics Committee of the First Affiliated Hospital of Nanchang University. The A549 cells (2×10^6^ cells/200μl) were respectively injected subcutaneously into the left armpit of nude mice to establish the subcutaneous xenograft model. The subcutaneous xenograft was classified into five groups (control, IR, IR+RAPA, IR+SAHA, IR+RAPA+SAHA, 5 mice/group). We observed the growth and diet of nude mice, and the volume of subcutaneous tumors, and the weight of nude mice were measured every four days. About 4 weeks later, all the nude mice were sacrificed. The tumors were weighed and fixed in 10% formalin.

### Statistical analysis

Data were expressed as means±SD and analyzed by analysis of variance (ANOVA). Statistical significance was determined using Student’s t-test for comparison between the means or one-way analysis of variance with post-hoc Dunnett’s test. Differences were considered significant at *p*<0.05. All analyses were performed using GraphPad Prism 8.3.0 software (San Diego, CA, USA).
